# Genome-wide association study revealed that the *TaGW8* gene was associated with kernel size in Chinese bread wheat

**DOI:** 10.1038/s41598-019-38570-2

**Published:** 2019-02-25

**Authors:** Xuefang Yan, Lei Zhao, Yan Ren, Zhongdong Dong, Dangqun Cui, Feng Chen

**Affiliations:** Agronomy College/National Key Laboratory of Wheat and Maize Crop Science, Henan Agricultural University, Zhengzhou, 450046 China

## Abstract

Using Wheat 90 K SNP assay, kernel-related traits of Chinese bread wheat were used to perform association mapping in 14 environments by GWAS. Results indicated that 996 and 953 of 4417 and 3172 significant SNPs for kernel length and thousand-kernel weight were located on the chromosome 7B. Haplotype analysis of these SNPs on 7B generated the block containing the predicted *TaGW8-B1* gene. *TaGW8-B1* gene was further cloned by sequencing in bread wheat and a 276-bp InDel was found in the first intron. *TaGW8-B1* without and with the 276-bp InDel were designated as *TaGW8-B1a* and *TaGW8-B1b*, respectively. Analysis of agronomic traits indicated that cultivars with *TaGW8-B1a* possessed significantly wider kernel width, significantly more kernel number per spike, longer kernel length, higher thousand-kernel weight and more spikelet number per spike than cultivars with *TaGW8-B1b*. Furthermore, cultivars with *TaGW8-B1a* possessed significantly higher yield than cultivars with *TaGW8-B1b*. Therefore, *TaGW8-B1a* was considered as a potentially superior allele. Meanwhile, *TaGW8-B1a* possessed a significantly higher expression level than *TaGW8-B1b* in mature seeds by qRT-PCR. It possibly suggested that the high expression of *TaGW8-B1* was positively associated with kernel size in bread wheat. Distribution of *TaGW8-B1* allele indicated that *TaGW8-B1a* has been positively selected in Chinese wheat.

## Introduction

Bread wheat (*Triticum aestivum* L.) is one of the most important crops in the world. With the increasing population, continuous improvement of yield potential is the long-term goal of wheat breeding^1^. Three elements of wheat yield consist of thousand kernel weight (TKW), spike number per Mu (666.7 m^2^) and kernel number per spike. Of them, TKW is considered to have an important influence on yield and could be determined by kernel size. Among kernel size-related traits (e.g. kernel length, kernel width, kernel thickness etc.), kernel width shows the highest correlation with kernel weight^2^. To date, many yield-related genes have been identified or cloned in crops, such as *GW2*^3^*, GW3* and *TGW6*^4^, *GW7*^5^, *GW8*^6^, *GIF1*^7^, *GS5*^8^, *OsSPL13*^9^ in rice, and *ZmGS5* and *ZmBAK1-7* genes in maize^10^.

Hexaploid wheat has a lager genome size (≈17.9 Gb) when compared with rice (≈400 Mb) and maize (≈3 Gb), which limited cloning of related genes to some extent. However, many QTLs for kernel size have been identified in polyploidy wheat^11–15^. QTLs associated with kernel size have been identified on all of the wheat chromosomes^16^. Major QTLs for kernel length were detected on the chromosomes 1A, 1B, 2A, 2B, 2D,4B, 5A, 5B, 5D and 7D^17,18^ and QTLs for kernel width were detected on the chromosomes 1D, 2A, 2B, 2D, 3B, 4B, 5B, 6D and 7D^16,19,20^. Moreover, some yield-related genes also have been cloned in polyploidy wheat. It has been reported that *TaGS5* genes on the short arm of chromosome 3A and 3D were significantly associated with kernel width, TKW, plant height, spike length and pedicle length^21–23^. The *TaCYP78A3* gene of *CYP78A* family, encoding cytochrome *CYP78A3 P450*, was identified on 7AS, 7BS and 7DS, and silencing this gene may cause 11% (*P* < 0.01) decrease in wheat seed size^24^. *TaGW2* gene has been proven to be significantly correlated to wheat kernel width and weight, flowering and maturity^25,26^. The *TaSus2* (Sucrose synthase type II)^27^ gene on the second homologous chromosomes of wheat was significantly associated with TKW.

As a powerful tool to analyze the genetic architecture of complex traits, GWAS has been widely applied in rice, maize and *Arabidopsis thaliana*^28–33^. With the rapid development of high-density SNP arrays in hexaploid wheat, GWAS is sharply getting popular to be used for association mapping of many traits in bread wheat, e.g. abiotic stress resistance^34,35^, floret fertility^36^, yield-related traits^37^, kernel number per spike^38^, etc. Gao *et al*.^39^ identified yield-related locus *QTKW.caas-7AL* in all of the surveyed environments using a F_8_ recombinant inbred lines population. GWAS for agronomic traits in hexaploid and texaploid wheats indicated that some SNPs on 7A and 7B were significantly associated with kernel length in multiple environments^40,41^.

In this study, we have successfully obtained the *TaGW8-B1* gene by the combination of haplotype analysis of GWAS, gene identification in the interval of Aikang 58 genome database and gene cloning. We further analyzed association of *TaGW8-B1* alleles with agronomic traits as well as yield in multiple environments. Results showed that *TaGW8-B1a* was a relatively superior allele in view of agronomic traits. The aim of this study is to provide valuable information for improvement of wheat yield in Chinese bread wheat breeding program.

## Materials and Methods

### Plant materials and field trails

In this study, a total of 365 wheat cultivars and advanced lines were planted at the Zhengzhou Scientific Research and Education Center of Henan Agricultural University (N34.9°, E113.6°) during 2012–2013, 2013–2014, and 2014–2015 cropping seasons. These materials, composed of landraces, historical cultivars and current cultivars, were collections from more than 10 provinces of China and are playing/played the important role in wheat breeding program of the Yellow and Huai wheat region as released cultivars or backbone parents. According to their pedigrees, agronomic performances, cultivated areas and released regions, 163 of the 365 wheat cultivars were further selected to plant at the Zhengzhou, Zhumadian and Anyang during 2012–2013, 2013–2014, and 2014–2015 cropping seasons for genotyping.

The 246 very recent wheat cultivars or advanced lines were selected from the Winter Wheat Regional Trials in Henan province during 2013–2016 and were planted in 14 environments, including Xihua, Puyang, Zhoukou, Yanshi, Xuchang, Luohe, Wenxian, Xinxiang, Huaxian, Dancheng, Xiangcheng, Changyuan, Tongxu and Zhengzhou. Some of them have been released as cultivars in recent two years due to their superior performance in field. Each cultivar or advanced line was planted in a full plot containing 12 rows with 250 cm long row and 23 cm row space. This experiment was designed by a completely randomized block design with three replications. After fully matured, all wheat plants in the whole plot of each cultivar were harvested for measuring yield per plot and then the results were further converted into yield per Mu (666.7 m^2^).

The plant height, pedicle length, spike length and spikelet number of ten spikes marked with red ribbon for each accession surveyed were investigated and measured in the field before harvested. After all grain samples were dried under natural conditions, kernel number of ten spikes, 10-kernel length, 10-kernel width, grain length/grain width ratio and TKW of each cultivar were investigated, respectively. No lodge occurred in whole field experiment with the help of supporting net for landraces.

### Genotyping and genome-wide association study

The selected 163 wheat cultivars were genotyped with 90 K SNP assay^42^ as described in Sun *et al*.^41^. However, different from the method of Sun *et al*.^41^ for quality control, the all SNP chip data was only filtered by eliminating the SNP loci without allelic variation in all surveyed cultivars. The analysis of association between kernel-related traits and SNP loci was performed using mixed linear model considering relationship and population structure^43,44^. GWAS was performed using GAPIT packages in R for windows 3.3.1. The threshold for significance markers set at 1.0 e-3 for p value in order to analyze GWAS results among 14 environmental conditions^41^.

### Acquisition and analysis of candidate genes

The haplotype analysis of detected and clustered significant SNPs loci associated with kernel traits were performed with software Haploview 4.2. The blocks were generated by Haploview based on confidence intervals by Gabriel *et al*.^45^. Genes were achieved from gene annotation of unpublished database of Aikang 58 genome in the block.

### PCR amplification and primer designing

All genomic DNAs were rapidly extracted from wheat kernel based on the method of Chen *et al*.^46^. BioRad-T100 were used to amplify PCR amplifications. PCR reaction system and PCR products detection were implemented according the method of Wang *et al*.^21^. The expected target fragments of PCR products were recovered by SanPrep Column DNA Gel Extraction Kit (Shanghai Biological Technology Co., Ltd.). The purified products were ligated with PMD19-T vector (TaKaRa Biotechnology Co., Ltd., Dalian) and then were transformed into *E.coli* DH-5*α* competent cells. The bacteria solutions containing the target fragments were sequenced by Shanghai Sangong Biotech Co., Ltd. after identification by bacteria liquid PCR. Five sub-clones of each sample were sent out for sequencing from both directions.

Six primer sets (TaGW8-P2~TaGW8-P7) were designed to amplify *TaGW8-B1* genomic DNA sequence variations in Chinese wheat cultivar surveyed. All primers were designed by the software Premier Primer 3.0 (http://primer3.ut.ee/) and were synthesized by Shanghai Biological Technology Co., Ltd. Sequence alignment and assembling were carried out with the software DNAMAN (Version 6.0). The authenticity and reliability of the sequencing results were verified by Chromas (http://technelysium.com.au/wp) and FinchTV 1.5.0 (http://www.geospiza.com/Products/finchtv.shtml).

### Quantitative real-time PCR of *TaGW8-B1a* and *TaGW8-B1b* genotypes

The total RNA was extracted by Trizol reagent and was reverse-transcribed into cDNA with PrimeScript RT reagent kit contained gDNA eraser (TaKaRa Biotechnology Co. Ltd, Dalian, China). All operations were implemented according to the Kit instructions. The *β*-actin gene as reference was used to detect the expressions of *TaGW8-B1a* and *TaGW8-B1b* alleles using qRT-PCR (quantitative real-time PCR). The specific primer sets TaGW8-P8 was designed to test the expression levels of *TaGW8-B1a* (NCBI No. MK388407) and *TaGW8-B1b* (NCBI No. MK388408) alleles. cDNAs of the mature seeds of 10 cultivars (Xinmai 18, Shi 4185, Aikang 58, Songhuajiang 1, Shi 82-5448, Lainong 9217, Yumai 13, Yumai 56, Yunong 201 and Beinongda 6282) with *TaGW8-B1a* and 8 cultivars (Ying S 15, Shi 84-7111, Bainong 95(01)-1-A, Yunong 202, Huaimai 19, Yumai 58, Zhoumai 20, Bainong 3217) with *TaGW8-B1b* were extracted to perform qRT-PCR for comparison of relative expression levels of *TaGW8-B1a* and *TaGW8-B1b* alleles.

The iQ5 real-time PCR detection system (Bio-Rad, Richmond, CA) was used to perform Real-Time PCR Quantification. The PCR reactions consisted of the following cycling conditions: 95 °C for 2 min, 40 cycles of 95 °C for 10 s, 60 °C for 10 s, 72 °C for 20 s and final extension of 72 °C for 10 min. The data analysis process was used by 2^−ΔΔCT^ method.

### Statistical analysis

Correlation coefficients among agronomic traits surveyed in this study were calculated by software Excel 2013. A one-way analysis of variance (ANOVA) using the SPSS 19.0 statistical software and Duncan’s multiple range test (DMRT) was used to identify significant (p < 0.05) differences between group averages.

## Results

### Overview the GWAS of kernel traits

**Figure 1:**
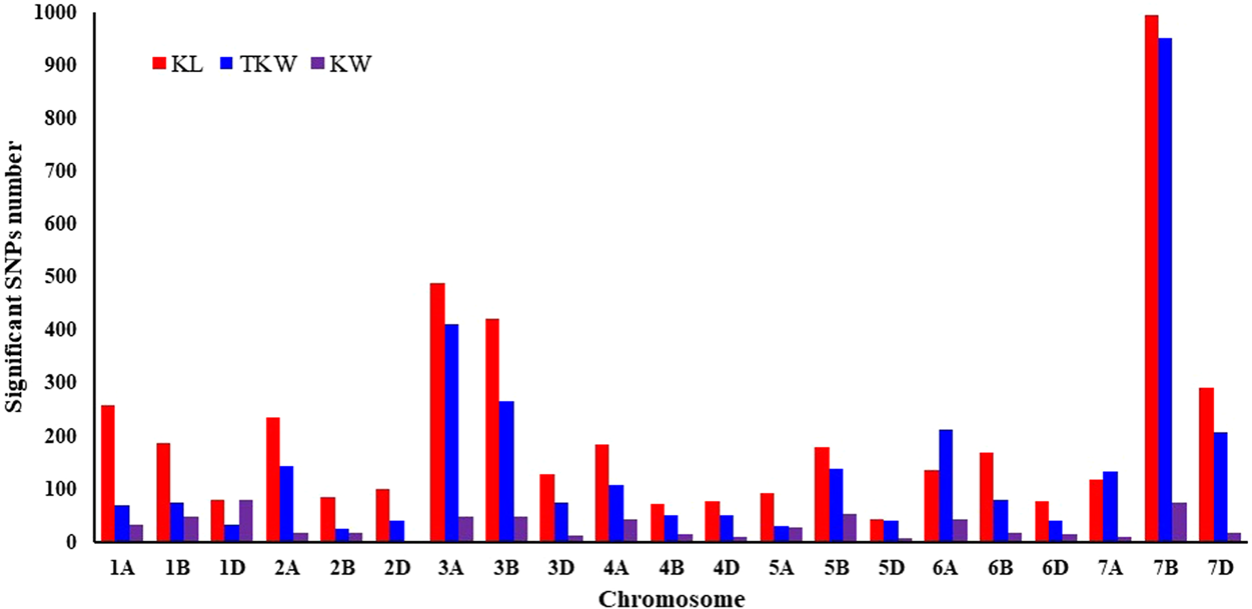
Distribution of significant SNPs for traits related to kernel size on the wheat chromosomes by GWAS.

After excluding poor quality data, 44,791 polymorphism SNPs was used for GWAS and results showed that a total of 4417, 3172 and 650 SNPs were identified to be significantly associated with kernel length, TKW and kernel width, respectively, and they distributed on all chromosomes. Further analysis indicated that 996 (22.5%) SNPs for kernel length, 953 (30.0%) SNPs for TKW and 75 (11.5%) SNPs for kernel width were located on the chromosome 7B (Fig. 1). Therefore, there are possibly some genes modulating kernel size on chromosome 7B.

### Further analysis of the significant loci associated with kernel trait

Compared to our previous study^41^, some new SNPs were detected to be significantly associated with kernel traits for more than four environments, e.g. 7 SNPs (wsnp_JD_c26552_21868492 on 6A, wsnp_Ex_c2426_4542393 on 2A, Kukri_c28695_269, Excalibur_c1845_4911, RAC875_c45115_509, Excalibur_c1845_718 and RAC875_c45115_340 on 1A) for kernel length, and 4 SNPs (BobWhite_c1059_1825 on 6D, wsnp_JD_c19925_17854742 and wsnp_Ku_c3929_7189422 on 7A, RAC875_c9457_457 on 1A) for TKW (Table 1). Further analysis of the significant SNPs on 7B for kernel length and TKW, 85 and 81 of the 996 (kernel length) and 953 (TKW) significant SNPs were detected in at least 4 environments and were designated as repetitively significant SNP.

**Table 1:** P values of T-test for some significant SNPs detected by GWAS.

Trait	SNP/Gene Name	Chr.	Allele	Number	Phenotype	2012–2013 AY-1	2012–2013 AY-2	2012–2013 ZZ-1	2012–2013 ZZ-2	2012–2013 ZMD-1	2012–2013 ZMD-2	2013–2014 AY-1	2013–2014 AY-2	2013–2014 ZZ-1	2013–2014 ZZ-2	2013–2014 ZMD-1	2013–2014 ZMD-2	2014–2015 ZZ-1	2014–2015 ZZ-2
KL	wsnp_JD_c26552_21868492	6A	CC/TT	89/62	7.20/6.87 cm	0.000	0.000	0.000	0.000	0.000	0.000	0.000	0.000	0.000	0.000	0.000	0.000	0.000	0.000
	wsnp_Ex_c2426_4542393	2A	CC/TT	67/73	7.23/6.93 cm	0.001	0.001	0.000	0.000	0.000	0.000	0.000	0.000	0.000	0.000	0.000	0.000	0.000	0.006
	Kukri_c28695_269	1A	AA/GG	14/146	6.65/7.10 cm	0.002	0.005	0.002	0.000	0.000	0.000	0.000	0.000	0.000	0.000	0.000	0.000	0.001	0.010
	Excalibur_c1845_4911	1A	CC/TT	146/14	7.10/6.65 cm	0.002	0.005	0.002	0.000	0.000	0.000	0.000	0.000	0.000	0.000	0.000	0.000	0.001	0.010
	RAC875_c45115_509	1A	AA/GG	14/146	6.65/7.10 cm	0.002	0.005	0.002	0.000	0.000	0.000	0.000	0.000	0.000	0.000	0.000	0.000	0.001	0.010
	Excalibur_c1845_718	1A	CC/TT	146/14	7.10/6.65 cm	0.002	0.005	0.002	0.000	0.000	0.000	0.000	0.000	0.000	0.000	0.000	0.000	0.001	0.010
	RAC875_c45115_340	1A	GG/TT	16/146	6.60/7.10 cm	0.001	0.002	0.001	0.000	0.000	0.000	0.000	0.000	0.000	0.000	0.000	0.000	0.002	0.016
TKW	BobWhite_c1059_1825	6D	GA/AA	134/27	48.36/52.09 g	0.000	0.001	0.007	0.005	0.005	0.001	0.000	0.000	0.000	0.000	0.001	0.001	0.004	0.131
	wsnp_JD_c19925_17854742	7A	TC/CC	147/13	48.46/54.62 g	0.000	0.000	0.004	0.001	0.001	0.000	0.000	0.000	0.000	0.000	0.000	0.000	0.000	0.010
	wsnp_Ku_c3929_7189422	7A	CC/CT	12/105	54.10/48.85 g	0.002	0.003	0.022	0.016	0.002	0.003	0.000	0.000	0.003	0.001	0.000	0.001	0.000	0.032
	RAC875_c9457_457	1A	CC/TC	69/70	50.06/47.68 g	0.004	0.004	0.003	0.020	0.000	0.000	0.048	0.082	0.007	0.026	0.010	0.010	0.004	0.092

**Figure 2:**
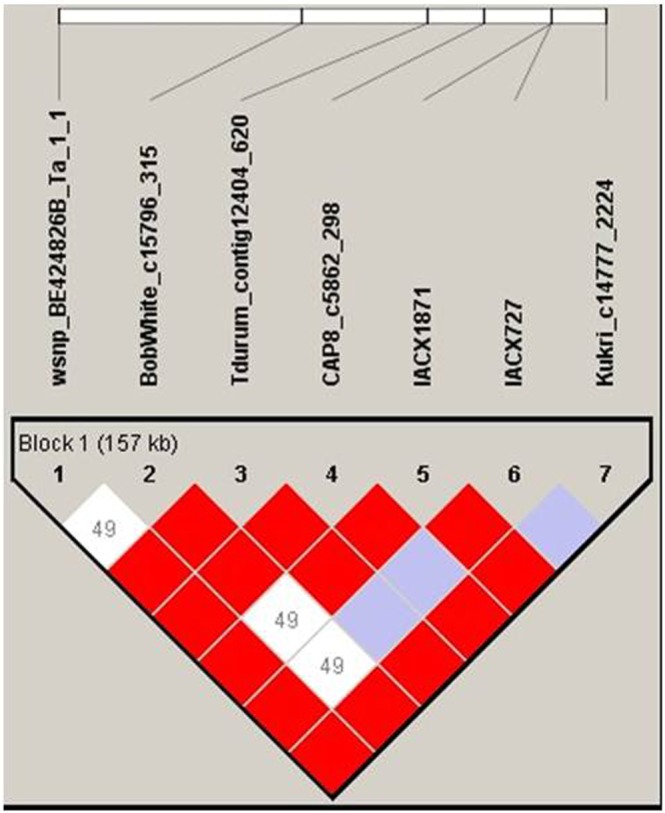
Haplotype analysis of significant SNPs on 7BS. The color represents the linkage between SNPs, and the deeper color means the higher linkage between SNPs.

As the repetitively significant SNPs are densely distributed on 7BS, haplotype analysis of significant SNPs on 7BS showed that seven significant SNPs for kernel length were mapped in a same block (Fig. 2), i.e. wsnp_BE424826B_Ta_1_1, BobWhite_c15796_315, IACX727, IACX1871, CAP8_c5862_298, Kukri_c14777_2224 and Tdurum_contig12404_620 (Table 2). Meanwhile, six of the seven SNPs (except for BobWhite_c15796_315) were also significantly associated with TKW. Blasting of the seven significant SNPs in Aikang 58 genome database (unpublished) indicated that this block between wsnp_BE424826B_Ta_1_1 and Tdurum_contig12404_620 ranged from 196781812 to 225448926 contained 28.667114 Mb (Table 2). According to the gene annotation of AK58 genome, the sequence between wsnp_BE424826B_Ta_1_1 and Tdurum_contig12404_620 contained 247 genes. Of the 247 genes, the annotated gene ranging from 219494761 to 219499586 is homologous to *OsGW8* (GenBank: JX867119.1) gene of rice by blasting in NCBI database.

**Table 2:** Significance SNPs located in one block for kernel length by GWAS.

SNP name	Allele	Starting position	Ending position
wsnp_BE424826B_Ta_1_1	T/G	196781812	196781932
BobWhite_c15796_315	T/C	200712177	200712277
IACX727	G/A	205129057	205129177
IACX1871	G/A	205129432	205129552
CAP8_c5862_298	T/C	217817649	217817549
Kukri_c14777_2224	G/A	217989844	217989744
TaGW8		219494761	219499586
Tdurum_contig12404_620	G/T	225448826	225448926

### Cloning of *TaGW8-B1* gene in bread wheat

**Figure 3:**
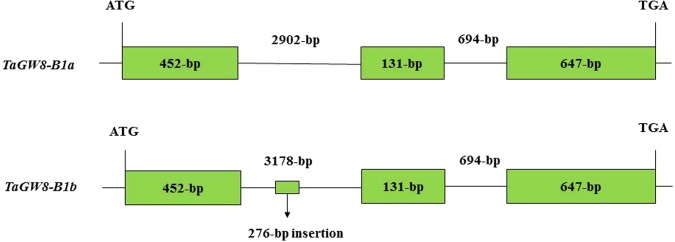
Schematic representation of *TaGW-B1a* and *TaGW-B1b* alleles in bread wheat.

According to the unpublished genomic sequence of Aikang 58, the primer set TaGW8-P1 (Table 3) was designed and a 1230-bp cDNA fragment containing full-length *TaGW8* cDNA sequence was successfully amplified with TaGW8-P1 in the cDNA of Aikamg58. Then six primer sets (TaGW8-P2~TaGW8-P7 in Table 3) were further designed to amplify *TaGW8* genomic sequence in Chinese bread cultivar. All successful PCR fragments with those primers were ligated into to the pGEM-T Easy vector and 15 subclones for each sample were successfully sequenced from both directions. Finally, a full-length *TaGW8* gDNA sequences were successfully assembled on B genomes of Aikang 58 (Fig. 3) and was designated as *TaGW8-B1* gene. Further analysis of gDNA sequences of *TaGW8-B1* gene with 4826 bp indicated that *TaGW8-B1* gene was composed of 3 exons and 2 introns (Fig. 3). The deduced amino acid sequence showed that the *TaGW8-B1* gene could encode a 409-aa protein with SBP (Squamosa-promoter Binding Protein. Pfam accession: 03110) domain at 105–179 interval as predicted in NCBI (https://www.ncbi.nlm.nih.gov/).

**Table 3:** Primer sets used for cloning and qRT-PCR of *TaGW8-B1* gene in this study.

Primer	Primer sequence (5′-3′)	Annealing Temp. (°C)	Size of PCR fragment (bp)
TaGW8-P1	Forward: CCAACACACAAGCTCCAGAC Reverse: GCAACACATGAACAGCCACT	58	1433
TaGW8-P2	Forward: GAATGAGAGGGACAGGGGAG Reverse: TCATGAATGGGGCCAGGATT	57	1151
TaGW8-P3	Forward: TCTCTTCGCTCATCCATTCCT Reverse: GCTATCCCCACAACATAGACC	54	1297
TaGW8-P4	Forward: ATGCAGTGTGGACTTTGTGG Reverse: TTGCGATGCCAGTTGAACAT	57	1149
TaGW8-P5	Forward: TCCTCTACTCGGAACCTGAA Reverse: GTGCCCATCAAGACGCTTTC	55	1235
TaGW8-P6	Forward: TGTCTAACTACCTAGGAGCATCT Reverse: TGTGAAGAAGGTAAAGAGTGGC	61	1073
TaGW8-P7	Forward: CTTGCAGCTTGTAACCCACC Reverse: GAACGTCGGTGCTCTGAAAT	61	1498
GW8-7B	Forward: CGCTCATCCATTCCTTCATCG Reverse: GCTATATGGGTTGGTGTCGC	60	1097/1373
TaGW8-P8	Forward: TTCTCTGATGGGCTGACTCC Reverse: TGGGGATGTGTTCAGTCTGT	60	121
*β*-actin gene primer	Forward: GTGTCGCACCAGAGGATCAT Reverse: CGCTGGCATACAAGGACAGA	60	153

### Molecular characterization of *TaGW8-B1* gene in bread wheat from the Yellow and Huai wheat region

**Figure 4:**
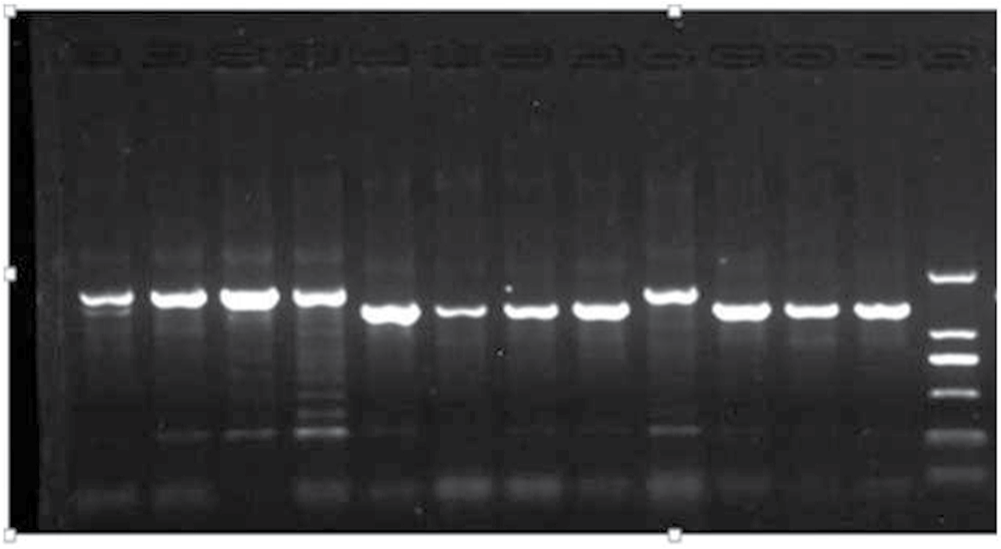
Identification of *TaGW-B1a and TaGW-B1b* alleles by GW7B markers in Chinese wheat cultivars. The 1097-bp fragment for *TaGW8-B1a* allele and the 1373-bp fragment for *TaGW8-B1b* allele. From left to right: Yumai 2, Xinyang 12, Taikong 6, Teng 15, SW625, Han 97–5085, Han 98-6026, SW652, Jinan 4, Beijing 6, Zhongmai 9, Jinan 13. DNA ladder is DL2000, including 2000-bp, 1000-bp, 750-bp, 500-bp, 250-bp, 100-bp fragments.

Based on the full-length *TaGW8-B1* genomic DNA sequence of Aikang58, six (TaGw8-P2~TaGw8-P7) primer sets were designed to identify allelic variation of *TaGW8-B1* gene in the selected 48 wheat cultivars with different kernel size and TKW. Finally, a nearly 300-bp InDel was identified in some cultivars when amplified with primer set TaGW8-P3. Sequencing results revealed that a 276-bp fragment was inserted in first intron at the position -1279 bp of the *TaGW8-B1* gene. The *TaGW8-B1* allele without the 276-bp InDel was designated as *TaGW8-B1a* and the *TaGW8-B1* allele with 276-bp InDel was designated as *TaGW8-B1b*. In addition, 4 cultivars including Songhuajiang 1, Xinong 164, Aikang 58 and Yannong 15 were further selected to sequence full length of *TaGW8-B1* sequence at the DNA level and no any difference was found in their exon region. Based on the 276-bp InDel, a new marker TaGW8-7B was developed to distinguish *TaGW8-B1a* and *TaGW8-B1b* alleles. It could generate a 1097-bp fragment for *TaGW8-B1a* allele and a 1373-bp fragment for *TaGW8-B1b* allele (Fig. 4) in bread wheat. Identification of *TaGW8-B1* allele by the marker TaGW8-7B indicated that 332 out of 365 cultivars from the Yellow and Huai wheat region possessed *TaGW8-B1a* allele and the remaining 33 cultivars possessed *TaGW8-B1b* allele. Based on BLASTn searches in database of Chinese Spring (https://urgi.versailles.inra.fr/blast/), the 276-bp InDel sequence in the *TaGW8-B1* gene showed more than 95% sequence similarity with more than 150 sequences distributing on all chromosomes in Chinese Spring genome. Furthermore, blasting the 276-bp sequence in NCBI database (https://blast.ncbi.nlm.nih.gov/) indicated that this sequence showed the 98% similarity with the *Triticum aestivum* transposon TREP 3040_Harbinger (Sequence ID: JF946486.1) and *Triticum aestivum* retrotransposons Gypsy TREP 3245_Sabrina (Sequence ID: JF946485.1). It suggested that the 276-bp InDel in the first intron of the *TaGW8-B1* gene was a transposon.

### Association of *TaGW8-B1* alleles with agronomic traits in Chinese bread wheat cultivars

Overview of coefficient correlation among different agronomic traits over three years in Chinese bread wheat cultivars (Supplementary Table S1) indicated that TKW showed extremely significant positive correlation with kernel width (*r* = 0.69**) and kernel length (*r* = 0.48**), and showed extremely significant negative correlation with kernel number per spike (*r* = −0.36**) (Supplementary Table S1). The result showed that grain size had a great influence on TKW but improving TKW possibly resulted in decrease of kernel number per spike in wheat breeding program.

Due to easy lodging of landraces, 329 historical and current cultivars were selected to analyze association of *TaGW8-B1a* and *TaGW8-B1b* alleles with agronomic traits over three years (Supplementary Table S2). Results indicated that cultivars with *TaGW8-B1a* allele possessed significantly wider kernel width (3.30 cm in 2013 and 3.58 cm in 2014) and more kernel number per spike (48.42 in 2015) than cultivars with *TaGW8-B1b* allele (3.22 cm in 2013 and 3.46 cm in 2014 cm for kernel width; 44.69 in 2015 for kernel number per spike) in 2015 (*P* < 0.05). In addition, cultivars with *TaGW8-B1a* allele also showed relatively higher TKW, longer kernel length and more spikelet number per spike than cultivars with *TaGW8-B1b* allele over three years even though these differences have not reached significant level (Table 4). Moreover, kernel length/kernel width ratio in Chinese historical and current cultivars with *TaGW8-B1b* allele was slightly higher than cultivar with *TaGW8-B1a* over three years. However, plant height, spike length and pedicle length did not show any significant difference between cultivars with *TaGW8-B1a* and *TaGW8-B1b* alleles over three years. Therefore, it suggested that the 276-bp InDel in the first intron of *TaGW8-B1b* gene contributed to a smaller kernel size in Chinese historical and current cultivars surveyed.

**Table 4:** Comparison of agronomic traits of cultivars with *TaGW8-B1a* and *TaGW8-B1b* alleles in Chinese current cultivars.

Trait	2012–2013		2013–2014		2014–2015	
	*TaGW8-B1a*	*TaGW8-B1b*	*TaGW8-B1a*	*TaGW8-B1b*	*TaGW8-B1a*	*TaGW8-B1b*
Sample number	298	31	298	31	298	31
Kernel length (mm)	6.88 ± 0.03a	6.79 ± 0.10a	6.96 ± 0.03a	6.83 ± 0.10a	6.88 ± 0.03a	6.83 ± 0.10a
Kernel width (mm)	3.30 ± 0.01a	3.22 ± 0.03b	3.58 ± 0.01a	3.46 ± 0.05b	3.42 ± 0.01a	3.36 ± 0.04a
Kernel length/kernel width ratio	2.09 ± 0.01a	2.12 ± 0.04a	1.95 ± 0.01a	1.97 ± 0.05a	2.02 ± 0.01a	2.04 ± 0.04a
Thousand-kernel weight (g)	43.21 ± 0.40a	41.07 ± 1.09a	50.98 ± 0.35a	50.19 ± 1.11a	42.95 ± 0.04a	42.31 ± 1.65a
Kernel number per spike	43.10 ± 0.61a	42.97 ± 3.60a	53.48 ± 0.65a	49.29 ± 2.36a	48.42 ± 0.57a	44.69 ± 1.42b
Plant height (cm)	72.38 ± 0.84a	76.88 ± 3.49a	78.40 ± 1.02a	75.23 ± 3.36a	91.80 ± 0.75a	95.91 ± 3.06a
Spike length (cm)	9.95 ± 0.09a	10.00 ± 0.41a	9.29 ± 0.10a	9.09 ± 0.34a	10.58 ± 0.10a	10.31 ± 0.30a
Pedicle length (cm)	24.87 ± 0.29a	25.49 ± 1.19a	30.12 ± 0.86a	27.38 ± 1.26a	30.40 ± 0.44a	30.13 ± 1.32a
Spikelet number per spike	19.29 ± 0.13a	19.17 ± 0.34a	19.88 ± 0.10a	19.33 ± 0.39a	19.29 ± 0.12a	19.19 ± 0.42a
Leaf length	NA	NA	20.91 ± 0.24a	20.94 ± 0.73a	24.00 ± 0.23a	23.87 ± 1.10a
Leaf width	NA	NA	1.90 ± 0.02a	1.91 ± 0.04a	1.94 ± 0.02a	1.86 ± 0.06a

Small letters after numbers showed significant (P < 0.05) differences.

**Figure 5:**
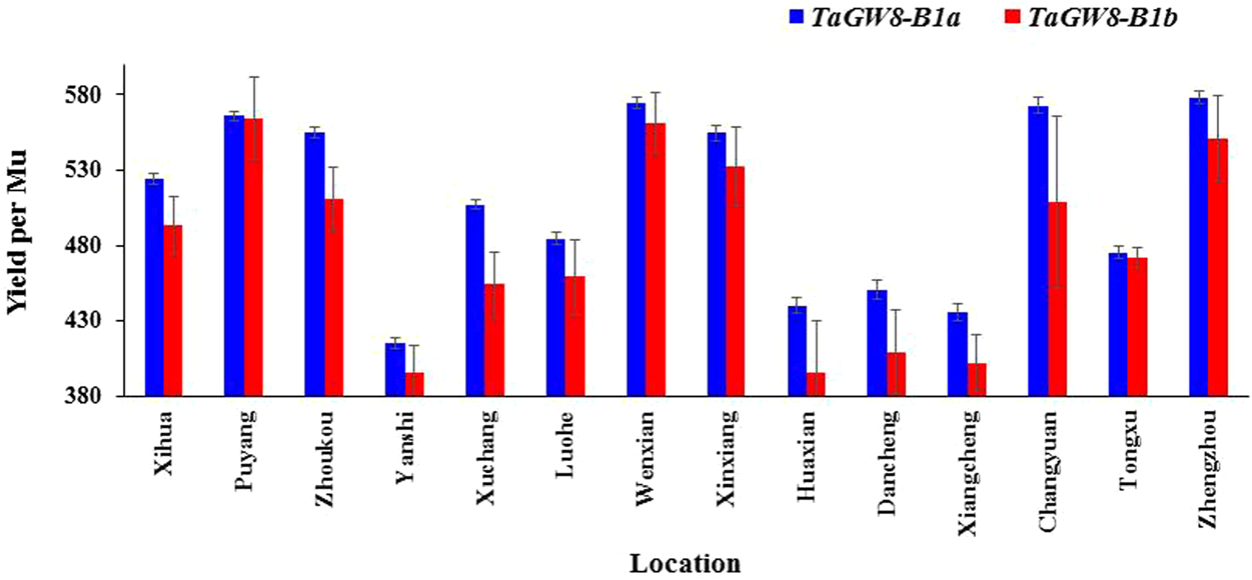
Comparison of yield per Mu of the surveyed 246 cultivars with *TaGW8-B1a* and *TaGW8-B1b* alleles in 14 locations.

Furthermore, the 246 recent wheat cultivars and advanced lines were used to evaluate influence of *TaGW8-B1a* and *TaGW8-B1b* alleles on wheat yield in 14 environments (Supplementary Table S2). Association of *TaGW8-B1a* and *TaGW8-B1b* alleles with yield indicated that cultivars with *TaGW8-B1a* allele possessed higher yield in all over 14 environments than cultivars with *TaGW8-B1b* allele (Fig. 5). In Changyuan and Xuchang, differences of yield were 63.85 kg and 52.84 kg per Mu between cultivars with *TaGW8-B1a* and *TaGW8-B1b* alleles. Therefore, *TaGW8-B1a* could be considered as a relative superior allele in view of agronomic traits including yield.

Additionally, distribution of *TaGW8-B1a* and *TaGW8-B1b* alleles indicated that there were 2 (5.88%) of 36 landraces, 8 (13.1%) of 69 early historical cultivars (before 1980s), 12 (14.3%) of 96 historical cultivars (1980s–2000s), 11 (15.1%) of 84 modern cultivars (After 2000s) possessing *TaGW8-B1b* allele. It showed that the inferior *TaGW8-B1b* allele was slightly increasing in cultivars from the Yellow and Huai wheat region but *TaGW8-B1a* allele was still predominant in all cultivars surveyed.

### Expression analysis of *TaGW8-B1a* and *TaGW8-B1b* genotypes

**Figure 6:**
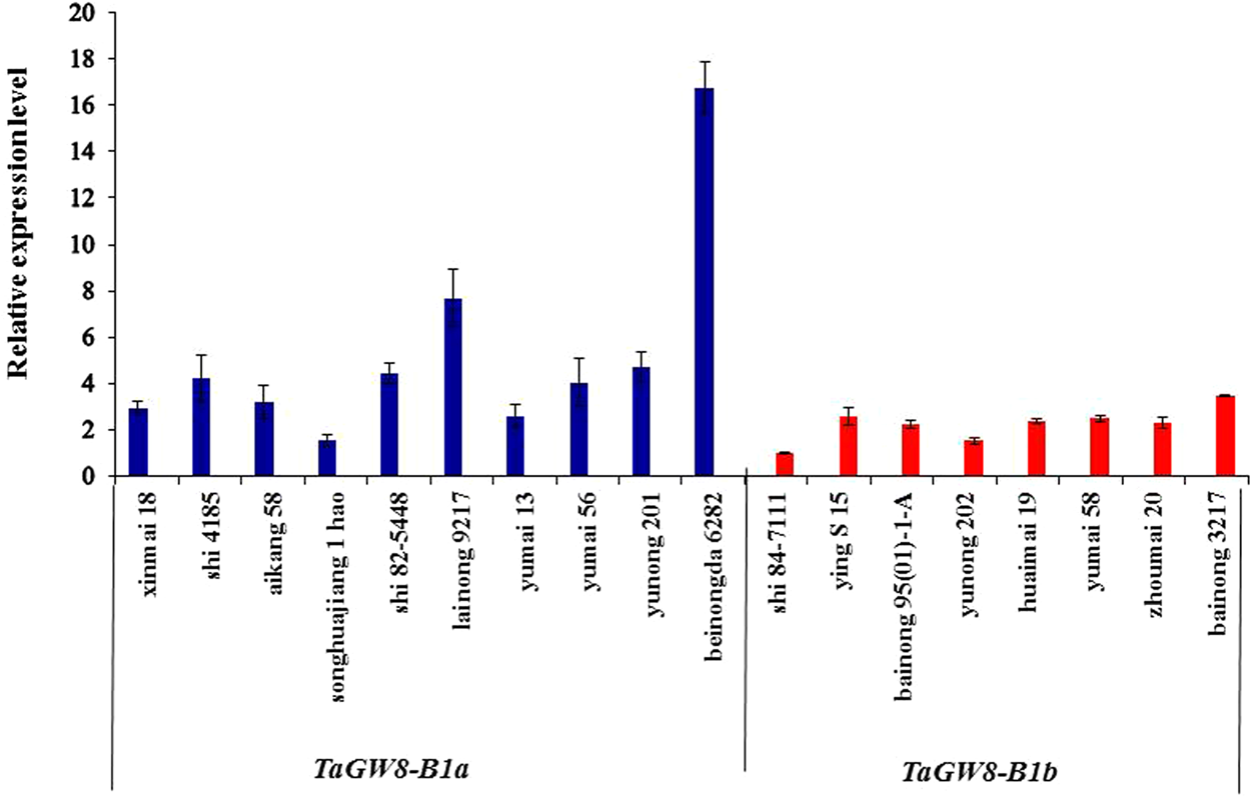
Relative expression levels of cultivars with *TaGW8-B1a* and *TaGW8-B1b* alleles in mature seeds.

Mature seeds of 18 Chinese current cultivars were selected to analyze relative expression levels of *TaGW8-B1a* and *TaGW8-B1b* alleles, and 10 and 8 out of the 18 cultivars belonged to the *TaGW8-B1a* and *TaGW8-B1b* alleles, respectively. qRT-PCR results indicated that averaged relative expression level of 10 cultivars with *TaGW8-B1a* were significantly higher than that of the 8 cultivars with *TaGW8-B1b* (Fig. 6). These results suggested that the higher expression of *TaGW8-B1* gene was possibly positively associated with wider kernel width and higher TKW.

## Discussion

Yield is one of the most important targets in bread wheat breeding program, whereas it has become more and more difficult for wheat breeders to significantly improve the wheat yield due to increasingly narrower genetic basis of wheat germplasms for wheat breeding in recent years. In this study, a *TaGW8-B1a* and *TaGW8-B1b* gene was identified to be associated with agronomic traits in bread wheat cultivars. Based on the 276-bp InDel in the first intron, *TaGW8-B1a* and *TaGW8-B1b* alleles were designated, and cultivars with *TaGW8-B1a* allele had wider kernel width, higher TKW, more spikelet number per spike and higher yield than cultivars with *TaGW8-B1b* allele. Therefore, *TaGW8-B1a* allele could be considered as a superior allele for wheat breeders in Chinese wheat breeding program.

Marker-assisted selection (MAS) is useful and efficient in wheat breeding^47^. Some agronomic traits-related relatively superior alleles including *TaSus2-2A-Hap-A*, *TaSus1-7B-Hap-T*, *TaGW2-6A-Hap-A*, *TaGW2-6B-Hap-1* and *TaGS5-A1a-b*^21–23,48,49^ have been reported to be associated with higher TKW and their corresponding markers could be used for improvement of agronomic traits in wheat breeding by marker-assisted selection. In this study, TaGW8-7B marker was developed to distinguish *TaGW8-B1a* and *TaGW8-B1b* alleles. Therefore, TaGW8-7B marker could be potentially combined with other preferred alleles (e.g. *TaSus2-2A-Hap-A*, *TaSus1-7B-Hap-T*, *TaGW2-6A-Hap-A*, *TaGW2-6B-Hap-1* and *TaGS5-A1a-b* etc.) to select a relatively ideal wheat lines in wheat breeding program. Meanwhile, there are many yield-related genes/QTLs previously reported on chromosome 7B including *TaSus1-7B* loci for high TKW^48^, *Hkps/sn-7B* loci for increasing kernels per spike and spikelet number per spike^50^, and *TaCYP78A3* gene for kernel size^24^ etc. Haplotypes *TaSus1-7B_Hap-T* and *Hkps/sn-7B-2* as well as overexpression of *TaCYP78A3* are preferred in view of improvement of yield-related traits according to previous reports^24,48,50^. However, some of them may be in repulsion to the *TaGW8-B1a* allele. Therefore, combination of *TaGW8-B1a* allele and other superior alleles at 7B loci should be considered for improvement of yield-related traits in wheat breeding program.

Previous work indicated that higher expression levels of *TaGS5-A1b* and *TaGS5-A1a-b* were associated with larger kernel size and higher TKW^21–23^. In this study, *TaGW8-B1a* showed higher relative expression levels than *TaGW8-B1b* in the mature seeds. Therefore, the high expression of *TaGW8-B1* possibly resulted in larger kernel size and higher yield but more work needs to be performed to further confirm this result.

Introns are non-coding sequences and thus was generally considered as non-functional region in gene regulation. However, some intronic sequences have unexpected function on transcription initiation and downstream regulatory element^51^. For example, the first introns of rice *α*-tubulin isotypes (Ostua1, Ostua2 and Ostua3) was a key regulatory element, *α*-tubulin gene family of rice sustained high level of marker-gene expression with rice α-tubulin first introns^52^. According to post-transcriptional mechanism, the first intron of the PhADF1 gene altered tissue-specific expression and the intron-mediated enhancement regulating the expression of the petunia was a conserved mechanism^53^. A 424-bp insertion in the first intron of *VRN-A1f-del/ins* leaded to spring habit in *T. timopheevii*^54^. A SNP change at GRP2 protein binding site in the first intron of *VRN-A1* gene was intimately related to the requirement of winter wheat vernalization^55^. Retrotransposon *SORE-1* insertion in the first intron resulted in attenuation of *FT2a* gene expression^56^. The gene expression influenced by intron also have been reported in other plants, such as sucrose transporter LeSUT1 of tomato^57^ (Weise *et al*. 2008) and the intron of Arabidopsis PRF2^53^. The study also showed that the role of intron in gene regulation was gradually weakening with increasing distance from the ATG^58^. In this study, the 276-bp InDel in first intron led to change of expression level of *TaGW8-B1* gene and thus resulted in cultivars possessing narrower kernel width, less spikelet number per spike, relatively lower TKW, shorter kernel length. It suggests that the 276-bp InDel in the first intron could alter the function of *TaGW8-B1* gene. However, the mechanism of intron affecting gene expression is still elusive by now.

Transposable elements as genetic components and one of main forces driving genomic diversity can move around in genome^59^. Meanwhile, transposable elements through insertion, transposition, excision, ectopic recombination and chromosome breakage have important effects on gene structure and function. A lot of studies have been reported about the function of the transposable elements insertion^60,61^. For example, *Vrn-D1s* with an 844-bp DNA transposon insertion in first intron was considered as a potentially preferred allele in view of agronomic traits^62^. *CsaMLO8* allele with a non-autonomous class LTR retrotransposable element insertion caused its mRNA alternative splice in resistant powdery mildew cucumber, and mutation of *CsaMLO8* was loss of function and led to hypocotyl resistance to powdery mildew due to insertion of a transposable element^63^. *MADS-box* genes with transposable elements insertion in Arabidopsis showed an important influence on regulation of the insertion point^64^*. Wx-B1n* allele with a 2178-bp transposon insertion leaded to loss of its function^65^. In this study, the 276-bp InDel was transposon according to blasting results in NCBI and URGI databases. The cultivars with insertion of this transposon showed the changed agronomic traits and relative expression level, indicating that this transposon in the first intron of *TaGW8-B1a* was probably functional by alternative splice.

## Supplementary information

Table S1-2
