# Supplementary material for: Genome-wide association study revealed that the TaGW8 gene was associated with kernel size in Chinese bread wheat
Source: Sci Rep. 2019 Feb 25;9:2702. doi: 10.1038/s41598-019-38570-2 (PMC6389898; doi:10.1038/s41598-019-38570-2)
Supplement: Supplementary file 1 — Table S1-2 [file 41598_2019_38570_MOESM1_ESM.pdf]

Genome-wide association study revealed that the *TaGW8* gene was associated with kernel size in Chinese bread wheat

Xuefang Yan, Lei Zhao, Yan Ren, Zhongdong Dong, Dangqun Cui, Feng Chen *

Agronomy College / National Key Laboratory of Wheat and Maize Crop Science, Henan Agricultural University, Zhengzhou 450046, China

Supplementary Table S1. Correlation coefficient among agronomic traits in Chinese bread wheat

Traits	Kernel length	Kernel width	Kernel length/kernel width ratio	Thousand-kernel weight	Plant height	Panicle length	Pedicle length	Spikelet number per spike	Leaf length
Kernel width	0.30**								
Kernel length/kernel width ratio	0.74**	-0.34**							
Thousand-kernel weight	0.48**	0.69**	NS						
Plant height	NS	-0.21**	NS	-0.14*					
Panicle length	NS	-0.22**	0.15**	NS	0.18**				
Pedicle length	NS	-0.19**	0.14*	-0.15**	0.56**	0.12*			
Spikelet number per spike	NS	NS	NS	-0.13*	-0.15**	0.28**	-0.16**		
Leaf length	NS	-0.24**	0.19**	-0.20**	0.37**	0.35**	0.34**	0.22**	
Leaf width	NS	NS	NS	NS	-0.36**	0.29**	-0.17**	0.39**	0.14*
Kernel number per spike	-0.20**	-0.12*	NS	-0.36**	-0.24**	0.25**	-0.16**	0.56**	0.12*

Supplementary Table S2. Molecular characterization of TaGW8-B1a and TaGW8-B1b alleles in bread wheat

No.	Cultivar	Type	Allele
1	Xichang76-4	Modern cultivar	TaGW8-B1a
2	Xichang76-9	Modern cultivar	TaGW8-B1a
3	Huapeiai	Modern cultivar	TaGW8-B1a
4	Zaosui30	Modern cultivar	TaGW8-B1a
5	Lunong55055	Modern cultivar	TaGW8-B1a
6	Xinmai9987	Modern cultivar	TaGW8-B1a
7	Xinmai19	Modern cultivar	TaGW8-B1a
8	Ji874-109	Modern cultivar	TaGW8-B1a
9	Lainong9217	Modern cultivar	TaGW8-B1a
10	Han4589	Modern cultivar	TaGW8-B1a
11	Neixiang182	Modern cultivar	TaGW8-B1a
12	Ji923235	Modern cultivar	TaGW8-B1a
13	04Zhong70	Modern cultivar	TaGW8-B1a
14	Gaoyou503	Modern cultivar	TaGW8-B1a
15	Yuanfeng898	Modern cultivar	TaGW8-B1a
16	Jinmai50	Modern cultivar	TaGW8-B1a
17	Jinmai54	Modern cultivar	TaGW8-B1a
18	Yumai47	Modern cultivar	TaGW8-B1a
19	Jiaixiaomai1	Modern cultivar	TaGW8-B1a
20	Yunong949	Modern cultivar	TaGW8-B1a
21	Qingfeng1	Modern cultivar	TaGW8-B1a
22	Lumai15	Modern cultivar	TaGW8-B1a
23	Jinmai33	Modern cultivar	TaGW8-B1a
24	Jinmai47	Modern cultivar	TaGW8-B1a
25	BN160	Modern cultivar	TaGW8-B1a
26	Yumai13	Modern cultivar	TaGW8-B1a
27	Jimai36	Modern cultivar	TaGW8-B1a
28	Jimai41	Modern cultivar	TaGW8-B1a
29	Fengkang13	Modern cultivar	TaGW8-B1a
30	Shan160	Modern cultivar	TaGW8-B1a
31	Xiaoyan4	Modern cultivar	TaGW8-B1a
32	Lumai19	Modern cultivar	TaGW8-B1a
33	Jinmai49	Modern cultivar	TaGW8-B1a
34	Yumai10	Modern cultivar	TaGW8-B1a
35	Jimai30	Modern cultivar	TaGW8-B1a
36	Huabei187	Modern cultivar	TaGW8-B1a
37	Hanxuan3	Modern cultivar	TaGW8-B1a
38	Xinmai18	Modern cultivar	TaGW8-B1a
39	Yumai53	Modern cultivar	TaGW8-B1a
40	Neixiang184	Modern cultivar	TaGW8-B1a
41	Jinmai61	Modern cultivar	TaGW8-B1a
42	Zhoumai16	Modern cultivar	TaGW8-B1a
43	Yanzhan4110	Modern cultivar	TaGW8-B1a
44	Ji95-5219	Modern cultivar	TaGW8-B1a
45	Yunong9901	Modern cultivar	TaGW8-B1a
46	Xinmai11	Modern cultivar	TaGW8-B1a
47	Pumai9	Modern cultivar	TaGW8-B1a
48	Zhoumai18	Modern cultivar	TaGW8-B1a
49	Huaimai20	Modern cultivar	TaGW8-B1a
50	Kaimai14	Modern cultivar	TaGW8-B1a
51	Fanmai5	Modern cultivar	TaGW8-B1a
52	Lankaoaizao8	Modern cultivar	TaGW8-B1a

53	Biyumai	Modern cultivar	TaGW8-B1a
54	Shan229	Modern cultivar	TaGW8-B1a
55	Shannong7859	Modern cultivar	TaGW8-B1a
56	Changle5	Modern cultivar	TaGW8-B1a
57	Zhengzhou24	Modern cultivar	TaGW8-B1a
58	Yunong035	Modern cultivar	TaGW8-B1a
59	Shanghe6	Modern cultivar	TaGW8-B1a
60	Xifeng	Modern cultivar	TaGW8-B1a
61	Yangmai12	Modern cultivar	TaGW8-B1a
62	Yumai8	Modern cultivar	TaGW8-B1a
63	Heibao-2	Modern cultivar	TaGW8-B1a
64	Lankao90(6)52-24	Modern cultivar	TaGW8-B1a
65	Youmai2	Modern cultivar	TaGW8-B1a
66	Haocheng9411	Modern cultivar	TaGW8-B1a
67	Zhou91177	Modern cultivar	TaGW8-B1a
68	Xinmai3306	Modern cultivar	TaGW8-B1a
69	Xinmai9178	Modern cultivar	TaGW8-B1a
70	Xinmai3380	Modern cultivar	TaGW8-B1a
71	Shi4185	Modern cultivar	TaGW8-B1a
72	Zhenghua0840-3	Modern cultivar	TaGW8-B1a
73	Zhenghua3563	Modern cultivar	TaGW8-B1a
74	Xinmai20	Modern cultivar	TaGW8-B1a
75	Yan893xuan	Modern cultivar	TaGW8-B1a
76	Neijiang31	Modern cultivar	TaGW8-B1a
77	Lankao90(6)21-7	Modern cultivar	TaGW8-B1a
78	Lankao6	Modern cultivar	TaGW8-B1a
79	Bainong64	Modern cultivar	TaGW8-B1a
80	Laizhou953	Modern cultivar	TaGW8-B1a
81	Zhoumai17	Modern cultivar	TaGW8-B1a
82	Kaimai18	Modern cultivar	TaGW8-B1a
83	Jimai20	Modern cultivar	TaGW8-B1a
84	Zhengmai9023	Modern cultivar	TaGW8-B1a
85	Luohan2	Modern cultivar	TaGW8-B1a
86	Luohan3	Modern cultivar	TaGW8-B1a
87	Longmai157	Modern cultivar	TaGW8-B1a
88	Longmai862	Modern cultivar	TaGW8-B1a
89	Hemai026	Modern cultivar	TaGW8-B1a
90	Aikang58	Modern cultivar	TaGW8-B1a
91	Yumai57	Modern cultivar	TaGW8-B1a
92	Bonong5	Modern cultivar	TaGW8-B1a
93	Lumai23	Modern cultivar	TaGW8-B1a
94	Qianjinzao	Modern cultivar	TaGW8-B1a
95	Pindong34	Modern cultivar	TaGW8-B1a
96	Wuyimai	Modern cultivar	TaGW8-B1a
97	Xian83(104)-11zhong"S"	Modern cultivar	TaGW8-B1a
98	Hezuo2	Modern cultivar	TaGW8-B1a
99	Songhuajiang1	Modern cultivar	TaGW8-B1a
100	Jinshajiang1	Modern cultivar	TaGW8-B1a
101	Yuanzhu	Modern cultivar	TaGW8-B1a
102	Yunong4408	Modern cultivar	TaGW8-B1a
103	Xinong881	Modern cultivar	TaGW8-B1a
104	Fu9465	Modern cultivar	TaGW8-B1a
105	Yumai51	Modern cultivar	TaGW8-B1a

106	Ji5092	Modern cultivar	TaGW8-B1a
107	Jiai9	Modern cultivar	TaGW8-B1a
108	JiC522-9	Modern cultivar	TaGW8-B1a
109	JiC527-3	Modern cultivar	TaGW8-B1a
110	JiC524-7	Modern cultivar	TaGW8-B1a
111	Xinong85(47)	Modern cultivar	TaGW8-B1a
112	Yunong416	Modern cultivar	TaGW8-B1a
113	Zhoumai23	Modern cultivar	TaGW8-B1a
114	Yumai56	Modern cultivar	TaGW8-B1a
115	Yumai50	Modern cultivar	TaGW8-B1a
116	Yumai3	Modern cultivar	TaGW8-B1a
117	Yumai9	Modern cultivar	TaGW8-B1a
118	Zhoumai22	Modern cultivar	TaGW8-B1a
119	Yumai55	Modern cultivar	TaGW8-B1a
120	Yumai59	Modern cultivar	TaGW8-B1a
121	Yumai21	Modern cultivar	TaGW8-B1a
122	Yumai16	Modern cultivar	TaGW8-B1a
123	Fuyang936	Modern cultivar	TaGW8-B1a
124	Yumai14you	Modern cultivar	TaGW8-B1a
125	Ji95-6203	Modern cultivar	TaGW8-B1a
126	Yumai4	Modern cultivar	TaGW8-B1a
127	Yumai1	Modern cultivar	TaGW8-B1a
128	Xinong979	Modern cultivar	TaGW8-B1a
129	Yunong201	Modern cultivar	TaGW8-B1a
130	Zhengmai9694	Modern cultivar	TaGW8-B1a
131	Xinmai208	Modern cultivar	TaGW8-B1a
132	Yunong209	Modern cultivar	TaGW8-B1a
133	Mianyang86-11	Modern cultivar	TaGW8-B1a
134	Mianyang85-41	Modern cultivar	TaGW8-B1a
135	Chuan77-1293	Modern cultivar	TaGW8-B1a
136	Shuangji2	Modern cultivar	TaGW8-B1a
137	Shuangji4	Modern cultivar	TaGW8-B1a
138	Jingshuang16	Modern cultivar	TaGW8-B1a
139	Lumai14	Modern cultivar	TaGW8-B1a
140	Ji87guan739	Modern cultivar	TaGW8-B1a
141	Yumai18	Modern cultivar	TaGW8-B1a
142	Yanshi93(13)-1-1-0-1-1	Modern cultivar	TaGW8-B1a
143	Jingshuang2	Modern cultivar	TaGW8-B1a
144	Changnong339-5-1	Modern cultivar	TaGW8-B1a
145	Changwei18	Modern cultivar	TaGW8-B1a
146	SW607	Modern cultivar	TaGW8-B1a
147	Han99-6143	Modern cultivar	TaGW8-B1a
148	R25	Modern cultivar	TaGW8-B1a
149	Fu63	Modern cultivar	TaGW8-B1a
150	Yuanxie62	Modern cultivar	TaGW8-B1a
151	Jinan17	Modern cultivar	TaGW8-B1a
152	SW601	Modern cultivar	TaGW8-B1a
153	SW601	Modern cultivar	TaGW8-B1a
154	SW601	Modern cultivar	TaGW8-B1a
155	Neixiang237	Modern cultivar	TaGW8-B1a
156	Neixiang188	Modern cultivar	TaGW8-B1a
157	876	Modern cultivar	TaGW8-B1a
158	605	Modern cultivar	TaGW8-B1a

159	Xiaoyan54	Modern cultivar	TaGW8-B1a
160	Fengyou6	Modern cultivar	TaGW8-B1a
161	Zhongyou9507	Modern cultivar	TaGW8-B1a
162	Haocheng8901	Modern cultivar	TaGW8-B1a
163	R59	Modern cultivar	TaGW8-B1a
164	Heixiaomai	Modern cultivar	TaGW8-B1a
165	Xiaoyan6	Modern cultivar	TaGW8-B1a
166	Shan225	Modern cultivar	TaGW8-B1a
167	Shannong28	Modern cultivar	TaGW8-B1a
168	Xinong164	Modern cultivar	TaGW8-B1a
169	HP2-112	Modern cultivar	TaGW8-B1a
170	SW608	Modern cultivar	TaGW8-B1a
171	SW609	Modern cultivar	TaGW8-B1a
172	Han98-4025	Modern cultivar	TaGW8-B1a
173	NP209-1-2-1	Modern cultivar	TaGW8-B1a
174	Pingyang181	Modern cultivar	TaGW8-B1a
175	Jing411	Modern cultivar	TaGW8-B1a
176	Beijing8686	Modern cultivar	TaGW8-B1a
177	Xuzhou14	Modern cultivar	TaGW8-B1a
178	Yangmai4	Modern cultivar	TaGW8-B1a
179	Yangmai5	Modern cultivar	TaGW8-B1a
180	Shandong1870xi	Modern cultivar	TaGW8-B1a
181	Beijing837	Modern cultivar	TaGW8-B1a
182	SW604	Modern cultivar	TaGW8-B1a
183	SW625	Modern cultivar	TaGW8-B1a
184	Lu955159	Modern cultivar	TaGW8-B1a
185	Han97-5085	Modern cultivar	TaGW8-B1a
186	Han98-6026	Modern cultivar	TaGW8-B1a
187	Han94-8014	Modern cultivar	TaGW8-B1a
188	SW652	Modern cultivar	TaGW8-B1a
189	Beijing6	Modern cultivar	TaGW8-B1a
190	Weimai4	Modern cultivar	TaGW8-B1a
191	Zhongmai9	Modern cultivar	TaGW8-B1a
192	Jinan13	Modern cultivar	TaGW8-B1a
193	Beijing841	Modern cultivar	TaGW8-B1a
194	Jinan4	Modern cultivar	TaGW8-B1a
195	Zhengyin1	Modern cultivar	TaGW8-B1a
196	Yanda1817	Modern cultivar	TaGW8-B1a
197	Mianyang79-2	Modern cultivar	TaGW8-B1a
198	Sumai3	Modern cultivar	TaGW8-B1a
199	Yumai29	Modern cultivar	TaGW8-B1a
200	Shanhan8675	Modern cultivar	TaGW8-B1a
201	Fengkang8	Modern cultivar	TaGW8-B1a
202	Shi82-5448	Modern cultivar	TaGW8-B1a
203	Han93-4686	Modern cultivar	TaGW8-B1a
204	Jishen5099	Modern cultivar	TaGW8-B1a
205	Lankao5	Modern cultivar	TaGW8-B1a
206	Han94-5316	Modern cultivar	TaGW8-B1a
207	Shannong253	Modern cultivar	TaGW8-B1a
208	89150	Modern cultivar	TaGW8-B1a
209	Youxuan14xuan-1	Modern cultivar	TaGW8-B1a
210	RS9701	Modern cultivar	TaGW8-B1a
211	Shan354	Modern cultivar	TaGW8-B1a

212	Han97-5297	Modern cultivar	TaGW8-B1a
213	Lankao4	Modern cultivar	TaGW8-B1a
214	Xu9639	Modern cultivar	TaGW8-B1a
215	Jimai6	Modern cultivar	TaGW8-B1a
216	01-Zhong424	Modern cultivar	TaGW8-B1a
217	01-Zhong427	Modern cultivar	TaGW8-B1a
218	Yanyou361	Modern cultivar	TaGW8-B1a
219	Zhongyou9507(ai)	Modern cultivar	TaGW8-B1a
220	Zhengmai98	Modern cultivar	TaGW8-B1a
221	9783	Modern cultivar	TaGW8-B1a
222	Zhengmai9201	Modern cultivar	TaGW8-B1a
223	Fengyou8	Modern cultivar	TaGW8-B1a
224	Linkang5027	Modern cultivar	TaGW8-B1a
225	Henong2552	Modern cultivar	TaGW8-B1a
226	Kang4285	Modern cultivar	TaGW8-B1a
227	Chuanyu10	Modern cultivar	TaGW8-B1a
228	Xingmai1	Modern cultivar	TaGW8-B1a
229	Yanzhan1	Modern cultivar	TaGW8-B1a
230	92C11-4-1-13-3-11	Modern cultivar	TaGW8-B1a
231	96C21-67-2-28	Modern cultivar	TaGW8-B1a
232	Cang87-1	Modern cultivar	TaGW8-B1a
233	01-Shi88	Modern cultivar	TaGW8-B1a
234	R57	Modern cultivar	TaGW8-B1a
235	Zhengmai8998	Modern cultivar	TaGW8-B1a
236	Lankao1	Modern cultivar	TaGW8-B1a
237	Zhengda6	Modern cultivar	TaGW8-B1a
238	Nanda010-2776-2	Modern cultivar	TaGW8-B1a
239	Keyi26	Modern cultivar	TaGW8-B1a
240	Gao38	Modern cultivar	TaGW8-B1a
241	Anyang1	Modern cultivar	TaGW8-B1a
242	Chuanmai8	Modern cultivar	TaGW8-B1a
243	Yuandong107	Modern cultivar	TaGW8-B1a
244	Shan150(ck4)	Modern cultivar	TaGW8-B1a
245	Xianyangchaodasui	Modern cultivar	TaGW8-B1a
246	Guinong11	Modern cultivar	TaGW8-B1a
247	BeinongdaA43	Modern cultivar	TaGW8-B1a
248	Beinongda6282	Modern cultivar	TaGW8-B1a
249	Beinongda92	Modern cultivar	TaGW8-B1a
250	Ai2	Modern cultivar	TaGW8-B1a
251	Ai4	Modern cultivar	TaGW8-B1a
252	Nongda6282	Modern cultivar	TaGW8-B1a
253	Zhengzhou8761	Modern cultivar	TaGW8-B1a
254	Huaiyin9628	Modern cultivar	TaGW8-B1a
255	Shi012056*	Modern cultivar	TaGW8-B1a
256	Gaoyou22	Modern cultivar	TaGW8-B1a
257	Gaoyou25	Modern cultivar	TaGW8-B1a
258	Gaoyou28	Modern cultivar	TaGW8-B1a
259	Yanshi16	Modern cultivar	TaGW8-B1a
260	ShannongM12	Modern cultivar	TaGW8-B1a
261	ShannongM17	Modern cultivar	TaGW8-B1a
262	JiC524-1	Modern cultivar	TaGW8-B1a
263	Mianyang8640	Modern cultivar	TaGW8-B1a
264	Guinong16	Modern cultivar	TaGW8-B1a

265	Yumai43	Modern cultivar	TaGW8-B1a
266	Henong35-4-14	Modern cultivar	TaGW8-B1a
267	Mianyang8168-0-14	Modern cultivar	TaGW8-B1a
268	Wan798	Modern cultivar	TaGW8-B1a
269	Zhengzhou9405	Modern cultivar	TaGW8-B1a
270	Xinmai13	Modern cultivar	TaGW8-B1a
271	Bonong653	Modern cultivar	TaGW8-B1a
272	Shannong413863	Modern cultivar	TaGW8-B1a
273	Shan89150	Modern cultivar	TaGW8-B1a
274	Xian93991ai	Modern cultivar	TaGW8-B1a
275	Wansu9908	Modern cultivar	TaGW8-B1a
276	Xinmai9408*	Modern cultivar	TaGW8-B1a
277	Xuzhou954	Modern cultivar	TaGW8-B1a
278	Shannong972363	Modern cultivar	TaGW8-B1a
279	Zhengyou6	Modern cultivar	TaGW8-B1a
280	Jing2001*	Modern cultivar	TaGW8-B1a
281	Longmai977	Modern cultivar	TaGW8-B1a
282	Longmai908	Modern cultivar	TaGW8-B1a
283	98Shuijian-1*	Modern cultivar	TaGW8-B1a
284	Longmai135*	Modern cultivar	TaGW8-B1a
285	Longmai328*	Modern cultivar	TaGW8-B1a
286	Xiaoyan81*	Modern cultivar	TaGW8-B1a
287	Beinong95	Modern cultivar	TaGW8-B1a
288	Jinan124	Modern cultivar	TaGW8-B1a
289	Jimai1	Modern cultivar	TaGW8-B1a
290	Yumai35	Modern cultivar	TaGW8-B1a
291	Jinmai45	Modern cultivar	TaGW8-B1a
292	Xian8	Modern cultivar	TaGW8-B1a
293	Yangmai7	Modern cultivar	TaGW8-B1a
294	Aisheng3	Modern cultivar	TaGW8-B1a
295	Yangmai6	Modern cultivar	TaGW8-B1a
296	Jimai2	Modern cultivar	TaGW8-B1a
297	Hao9468	Modern cultivar	TaGW8-B1a
298	Hesheng2	Modern cultivar	TaGW8-B1a
299	Shi84-7111	Modern cultivar	TaGW8-B1b
300	Wenmai6	Modern cultivar	TaGW8-B1b
301	Jimai26	Modern cultivar	TaGW8-B1b
302	Zhengmai004	Modern cultivar	TaGW8-B1b
303	Taikong6	Modern cultivar	TaGW8-B1b
304	Jimai32	Modern cultivar	TaGW8-B1b
305	TengS15	Modern cultivar	TaGW8-B1b
306	Bainong95（01）-1-A	Modern cultivar	TaGW8-B1b
307	Yunong202	Modern cultivar	TaGW8-B1b
308	Jimai38	Modern cultivar	TaGW8-B1b
309	Huaimai19	Modern cultivar	TaGW8-B1b
310	Yumai41	Modern cultivar	TaGW8-B1b
311	Aifeng3	Modern cultivar	TaGW8-B1b
312	Xinyang12	Modern cultivar	TaGW8-B1b
313	Yumai58	Modern cultivar	TaGW8-B1b
314	Yumai52	Modern cultivar	TaGW8-B1b
315	Zhoumai20	Modern cultivar	TaGW8-B1b
316	Yuan8444	Modern cultivar	TaGW8-B1b
317	Chang133	Modern cultivar	TaGW8-B1b

318	Yumai2	Modern cultivar	TaGW8-B1b
319	Bainong3217	Modern cultivar	TaGW8-B1b
320	Pinchun16	Modern cultivar	TaGW8-B1b
321	Ningchun10	Modern cultivar	TaGW8-B1b
322	Xinyin13-2	Modern cultivar	TaGW8-B1b
323	2820258	Modern cultivar	TaGW8-B1b
324	Zhengzhou005	Modern cultivar	TaGW8-B1b
325	Nannongda96Co76	Modern cultivar	TaGW8-B1b
326	XJ1	Modern cultivar	TaGW8-B1b
327	YN3	Modern cultivar	TaGW8-B1b
328	JiC527-2	Modern cultivar	TaGW8-B1b
329	Jihan2	Modern cultivar	TaGW8-B1b
330	Chixiaomai	**Landrace**	TaGW8-B1a
331	Chiyacao	**Landrace**	TaGW8-B1a
332	Fengchan3	**Landrace**	TaGW8-B1a
333	Bima4	**Landrace**	TaGW8-B1a
334	Chadianhong	**Landrace**	TaGW8-B1a
335	Hongxiuzi	**Landrace**	TaGW8-B1a
336	Fuzhuang30	**Landrace**	TaGW8-B1a
337	Heputou	**Landrace**	TaGW8-B1a
338	Hongquanmang	**Landrace**	TaGW8-B1a
339	Huixianhong	**Landrace**	TaGW8-B1a
340	Fan6	**Landrace**	TaGW8-B1a
341	Jiangdongmen	**Landrace**	TaGW8-B1a
342	Qianjiaomai	**Landrace**	TaGW8-B1a
343	Youbao	**Landrace**	TaGW8-B1a
344	Shanqianmai	**Landrace**	TaGW8-B1a
345	Damuzhiai	**Landrace**	TaGW8-B1a
346	Aimengniu	**Landrace**	TaGW8-B1a
347	Zhongnong28	**Landrace**	TaGW8-B1a
348	Nanda2419	**Landrace**	TaGW8-B1a
349	Nongda311	**Landrace**	TaGW8-B1a
350	Afu	**Landrace**	TaGW8-B1a
351	Zaoyangmai	**Landrace**	TaGW8-B1a
352	Xiaobaimai(jing856)	**Landrace**	TaGW8-B1a
353	Shenglimai	**Landrace**	TaGW8-B1a
354	Abo	**Landrace**	TaGW8-B1a
355	Ourou	**Landrace**	TaGW8-B1a
356	Nonglin10	**Landrace**	TaGW8-B1a
357	Shuiyuan86	**Landrace**	TaGW8-B1a
358	Luofulin10	**Landrace**	TaGW8-B1a
359	Ganmai8	**Landrace**	TaGW8-B1a
360	Ailiduo	**Landrace**	TaGW8-B1a
361	Yannong15	**Landrace**	TaGW8-B1a
362	Neixiang5	**Landrace**	TaGW8-B1a
363	Beijing6	**Landrace**	TaGW8-B1a
364	Xinong6028	**Landrace**	TaGW8-B1b
365	Jingyang60	**Landrace**	TaGW8-B1b
366	Tianning18	Modern cultivar	TaGW8-B1a
367	Zhongxin18	Modern cultivar	TaGW8-B1a
368	Jiamai99	Modern cultivar	TaGW8-B1a
369	Keyu368	Modern cultivar	TaGW8-B1a
370	Zhongmai108	Modern cultivar	TaGW8-B1a

371	Bainong1309	Modern cultivar	TaGW8-B1a
372	Hemai181	Modern cultivar	TaGW8-B1a
373	Taixue30	Modern cultivar	TaGW8-B1a
374	Qunximai11	Modern cultivar	TaGW8-B1a
375	Fengtian18	Modern cultivar	TaGW8-B1a
376	Heyu1	Modern cultivar	TaGW8-B1a
377	Shengmai102	Modern cultivar	TaGW8-B1a
378	Xinmai68	Modern cultivar	TaGW8-B1a
379	Zhengmai1869	Modern cultivar	TaGW8-B1a
380	Hongmai618	Modern cultivar	TaGW8-B1a
381	Fengdecunmai20	Modern cultivar	TaGW8-B1a
382	Xinyoumai2	Modern cultivar	TaGW8-B1a
383	Fanmai533	Modern cultivar	TaGW8-B1a
384	Xingnong168	Modern cultivar	TaGW8-B1a
385	Yanmai9719	Modern cultivar	TaGW8-B1a
386	Luo1807	Modern cultivar	TaGW8-B1a
387	Yanfeng712	Modern cultivar	TaGW8-B1a
388	Zimai627	Modern cultivar	TaGW8-B1a
389	Yunong805	Modern cultivar	TaGW8-B1a
390	Zhengxin758	Modern cultivar	TaGW8-B1a
391	Gengmai256	Modern cultivar	TaGW8-B1a
392	Fumai188	Modern cultivar	TaGW8-B1a
393	Lunxuan162	Modern cultivar	TaGW8-B1a
394	Jingjiumai11	Modern cultivar	TaGW8-B1a
395	Yunong804	Modern cultivar	TaGW8-B1a
396	Gengmai237	Modern cultivar	TaGW8-B1a
397	Jinmai1	Modern cultivar	TaGW8-B1a
398	Liming28	Modern cultivar	TaGW8-B1a
399	Qinmai158	Modern cultivar	TaGW8-B1a
400	Junmai118	Modern cultivar	TaGW8-B1a
401	Caiyuan1	Modern cultivar	TaGW8-B1a
402	Junhe183	Modern cultivar	TaGW8-B1a
403	Ximai505	Modern cultivar	TaGW8-B1a
404	Kaimai26	Modern cultivar	TaGW8-B1a
405	Zhoukang918	Modern cultivar	TaGW8-B1a
406	Zhengnong06118	Modern cultivar	TaGW8-B1a
407	Nongda399	Modern cultivar	TaGW8-B1a
408	Zhongying012	Modern cultivar	TaGW8-B1a
409	Zhouyumai36	Modern cultivar	TaGW8-B1a
410	Jinying18	Modern cultivar	TaGW8-B1a
411	Zhengmai082	Modern cultivar	TaGW8-B1a
412	Zhengmai516	Modern cultivar	TaGW8-B1a
413	Xuke877	Modern cultivar	TaGW8-B1a
414	Zhongkenmai7	Modern cultivar	TaGW8-B1a
415	Chuangxing6	Modern cultivar	TaGW8-B1a
416	Luyuan502	Modern cultivar	TaGW8-B1a
417	Zhongfengmai2	Modern cultivar	TaGW8-B1a
418	Hengmai18	Modern cultivar	TaGW8-B1a
419	Neimai6	Modern cultivar	TaGW8-B1a
420	Fengbao8	Modern cultivar	TaGW8-B1a
421	Tianhe6	Modern cultivar	TaGW8-B1a
422	Xinmai37	Modern cultivar	TaGW8-B1a
423	Yufeng702	Modern cultivar	TaGW8-B1a

424	Yushengmai119	Modern cultivar	TaGW8-B1a
425	Hemai6	Modern cultivar	TaGW8-B1a
426	Junmai667	Modern cultivar	TaGW8-B1a
427	Nongfeng111	Modern cultivar	TaGW8-B1a
428	Wenliang1	Modern cultivar	TaGW8-B1a
429	Pingan0602	Modern cultivar	TaGW8-B1a
430	Huayu3568	Modern cultivar	TaGW8-B1a
431	Guangtai369	Modern cultivar	TaGW8-B1a
432	Yunong169	Modern cultivar	TaGW8-B1a
433	Zhonglemai9	Modern cultivar	TaGW8-B1a
434	Jinsui8	Modern cultivar	TaGW8-B1a
435	Jingkemai6	Modern cultivar	TaGW8-B1a
436	Xinxuan17	Modern cultivar	TaGW8-B1a
437	TH161	Modern cultivar	TaGW8-B1a
438	Jinmai108	Modern cultivar	TaGW8-B1a
439	Jinyan5	Modern cultivar	TaGW8-B1a
440	Huayu126	Modern cultivar	TaGW8-B1a
441	Baiqiang1201	Modern cultivar	TaGW8-B1a
442	Yimai8	Modern cultivar	TaGW8-B1a
443	Hefeng3	Modern cultivar	TaGW8-B1a
444	Lunxuan163	Modern cultivar	TaGW8-B1a
445	Yunong019	Modern cultivar	TaGW8-B1a
446	Zhongle8	Modern cultivar	TaGW8-B1a
447	Ruisen218	Modern cultivar	TaGW8-B1a
448	Fannong3	Modern cultivar	TaGW8-B1a
449	Hemai2	Modern cultivar	TaGW8-B1a
450	Xuke732	Modern cultivar	TaGW8-B1a
451	Jinmai14	Modern cultivar	TaGW8-B1a
452	Haozhuangjia1	Modern cultivar	TaGW8-B1a
453	Xuyou46	Modern cultivar	TaGW8-B1a
454	Xumai457	Modern cultivar	TaGW8-B1a
455	Fengdecunmai19	Modern cultivar	TaGW8-B1a
456	Hangmai8	Modern cultivar	TaGW8-B1a
457	L668	Modern cultivar	TaGW8-B1a
458	Qiangmai29	Modern cultivar	TaGW8-B1a
459	Luyan260	Modern cultivar	TaGW8-B1a
460	Yanhao306	Modern cultivar	TaGW8-B1a
461	Caizhi204	Modern cultivar	TaGW8-B1a
462	Zhengpinmai24	Modern cultivar	TaGW8-B1a
463	Anyumai18	Modern cultivar	TaGW8-B1a
464	Wenyu019	Modern cultivar	TaGW8-B1a
465	Dapingyuan18	Modern cultivar	TaGW8-B1a
466	Jingyumai1	Modern cultivar	TaGW8-B1a
467	Luomai718	Modern cultivar	TaGW8-B1a
468	Yanmai26	Modern cultivar	TaGW8-B1a
469	Junsui188	Modern cultivar	TaGW8-B1a
470	Tianmai119	Modern cultivar	TaGW8-B1a
471	Fengmai53	Modern cultivar	TaGW8-B1a
472	Danmai108	Modern cultivar	TaGW8-B1a
473	Xinmai38	Modern cultivar	TaGW8-B1a
474	Changshengmai1	Modern cultivar	TaGW8-B1a
475	Defeng108	Modern cultivar	TaGW8-B1a
476	Shaomai25	Modern cultivar	TaGW8-B1a

477	Tongfeng736	Modern cultivar	TaGW8-B1a
478	Meng615	Modern cultivar	TaGW8-B1a
479	Boyu866	Modern cultivar	TaGW8-B1a
480	Jiamei8	Modern cultivar	TaGW8-B1a
481	Xianhong169	Modern cultivar	TaGW8-B1a
482	Yanmai888	Modern cultivar	TaGW8-B1a
483	Shengyuan928	Modern cultivar	TaGW8-B1a
484	Jun5366	Modern cultivar	TaGW8-B1a
485	Xuke682	Modern cultivar	TaGW8-B1a
486	Xinzhi519	Modern cultivar	TaGW8-B1a
487	Xuyan2	Modern cultivar	TaGW8-B1a
488	Hangmai6	Modern cultivar	TaGW8-B1a
489	Yulong1325	Modern cultivar	TaGW8-B1a
490	Fanmai536	Modern cultivar	TaGW8-B1a
491	Yufeng1366	Modern cultivar	TaGW8-B1a
492	Xinmai12	Modern cultivar	TaGW8-B1a
493	Xunong618	Modern cultivar	TaGW8-B1a
494	Ningnong718	Modern cultivar	TaGW8-B1a
495	Yuyan168	Modern cultivar	TaGW8-B1a
496	Shunmai8	Modern cultivar	TaGW8-B1a
497	Tunmai728	Modern cultivar	TaGW8-B1a
498	Aomai18	Modern cultivar	TaGW8-B1a
499	Xu331	Modern cultivar	TaGW8-B1a
500	Pumai27	Modern cultivar	TaGW8-B1a
501	Tianlaoda3	Modern cultivar	TaGW8-B1a
502	Xingyu7	Modern cultivar	TaGW8-B1a
503	Xinong18	Modern cultivar	TaGW8-B1a
504	Yongfengnong2	Modern cultivar	TaGW8-B1a
505	Guangtai213	Modern cultivar	TaGW8-B1a
506	Qiule2126	Modern cultivar	TaGW8-B1a
507	Nongfeng8210	Modern cultivar	TaGW8-B1a
508	Saidemai7	Modern cultivar	TaGW8-B1a
509	Dongfanghongmai6	Modern cultivar	TaGW8-B1a
510	Jinmai18	Modern cultivar	TaGW8-B1a
511	Jimai210	Modern cultivar	TaGW8-B1a
512	Kaimai27	Modern cultivar	TaGW8-B1a
513	Jinmai109	Modern cultivar	TaGW8-B1a
514	Hengda58	Modern cultivar	TaGW8-B1a
515	Maifeng9	Modern cultivar	TaGW8-B1a
516	Jinchengmai10	Modern cultivar	TaGW8-B1a
517	Xianmai15	Modern cultivar	TaGW8-B1a
518	Yanmai988	Modern cultivar	TaGW8-B1a
519	Xianyuan988	Modern cultivar	TaGW8-B1a
520	Lunxuan169	Modern cultivar	TaGW8-B1a
521	Xinmai8	Modern cultivar	TaGW8-B1a
522	Wenmai29	Modern cultivar	TaGW8-B1a
523	Wenyuan0528	Modern cultivar	TaGW8-B1a
524	Jinwoye1	Modern cultivar	TaGW8-B1a
525	Changmai13	Modern cultivar	TaGW8-B1a
526	Kun169	Modern cultivar	TaGW8-B1a
527	SM110	Modern cultivar	TaGW8-B1a
528	Bainong219	Modern cultivar	TaGW8-B1a
529	Sanhe1	Modern cultivar	TaGW8-B1a

530	Xizawuahao	Modern cultivar	TaGW8-B1a
531	Yufeng6	Modern cultivar	TaGW8-B1a
532	Dapingyuan007	Modern cultivar	TaGW8-B1a
533	Songmai518	Modern cultivar	TaGW8-B1a
534	Shengzhou209	Modern cultivar	TaGW8-B1a
535	Yunong99	Modern cultivar	TaGW8-B1a
536	Shangmai162	Modern cultivar	TaGW8-B1a
537	Junmai612	Modern cultivar	TaGW8-B1a
538	Jiyanmai10	Modern cultivar	TaGW8-B1a
539	Aifeng338	Modern cultivar	TaGW8-B1a
540	Fengmai52	Modern cultivar	TaGW8-B1a
541	Chuangmai11	Modern cultivar	TaGW8-B1a
542	Wanmai99	Modern cultivar	TaGW8-B1a
543	Xinhuamai818	Modern cultivar	TaGW8-B1a
544	Zhumai706	Modern cultivar	TaGW8-B1a
545	Taifeng11	Modern cultivar	TaGW8-B1a
546	Zhongmai10	Modern cultivar	TaGW8-B1a
547	Jinchengmai12	Modern cultivar	TaGW8-B1a
548	Chuangxin106	Modern cultivar	TaGW8-B1a
549	Huaichuan361	Modern cultivar	TaGW8-B1a
550	Xianmai522	Modern cultivar	TaGW8-B1a
551	Jinfeng216	Modern cultivar	TaGW8-B1a
552	Jinzhan638	Modern cultivar	TaGW8-B1a
553	Xiangmai1123	Modern cultivar	TaGW8-B1a
554	Lunxuan167	Modern cultivar	TaGW8-B1a
555	Xuke158	Modern cultivar	TaGW8-B1a
556	Chuangxin116	Modern cultivar	TaGW8-B1a
557	Zhengda101	Modern cultivar	TaGW8-B1a
558	Ximai329	Modern cultivar	TaGW8-B1a
559	Luomai166	Modern cultivar	TaGW8-B1a
560	Fannong1	Modern cultivar	TaGW8-B1a
561	Kelinmai969	Modern cultivar	TaGW8-B1a
562	Bomai118	Modern cultivar	TaGW8-B1a
563	Wohua066	Modern cultivar	TaGW8-B1a
564	Minfeng296	Modern cultivar	TaGW8-B1a
565	Zimai615	Modern cultivar	TaGW8-B1a
566	Xuanmai6	Modern cultivar	TaGW8-B1a
567	Weinong208	Modern cultivar	TaGW8-B1a
568	Yingmai182	Modern cultivar	TaGW8-B1a
569	Shenhua208	Modern cultivar	TaGW8-B1a
570	Jumai66	Modern cultivar	TaGW8-B1a
571	Yufeng2	Modern cultivar	TaGW8-B1a
572	Yumai117	Modern cultivar	TaGW8-B1a
573	LiangyuanA6	Modern cultivar	TaGW8-B1a
574	Zhenmai5	Modern cultivar	TaGW8-B1a
575	Xinxuan16	Modern cultivar	TaGW8-B1a
576	Lifu05	Modern cultivar	TaGW8-B1a
577	Fanyumai18	Modern cultivar	TaGW8-B1a
578	Yingman180	Modern cultivar	TaGW8-B1a
579	Xianmai521	Modern cultivar	TaGW8-B1a
580	Jiangmai816	Modern cultivar	TaGW8-B1a
581	Danmai118	Modern cultivar	TaGW8-B1a
582	Tianmin688	Modern cultivar	TaGW8-B1a

583	Shunmai299	Modern cultivar	TaGW8-B1a
584	Xuyan3	Modern cultivar	TaGW8-B1a
585	Zhengda3087	Modern cultivar	TaGW8-B1a
586	Zhaofeng668	Modern cultivar	TaGW8-B1a
587	Fengmai10	Modern cultivar	TaGW8-B1a
588	Chuangxing26	Modern cultivar	TaGW8-B1a
589	Yufeng1	Modern cultivar	TaGW8-B1a
590	Neile268	Modern cultivar	TaGW8-B1a
591	Pinghuayan3	Modern cultivar	TaGW8-B1a
592	Hongtaiyang2	Modern cultivar	TaGW8-B1a
593	Tianlaoda1	Modern cultivar	TaGW8-B1a
594	Xinyanmai98	Modern cultivar	TaGW8-B1a
595	Hongmai186	Modern cultivar	TaGW8-B1a
596	Huayan328	Modern cultivar	TaGW8-B1a
597	Huimai216	Modern cultivar	TaGW8-B1a
598	Nongda2018	Modern cultivar	TaGW8-B1a
599	Jiyanmai7	Modern cultivar	TaGW8-B1a
600	Jinfeng205	Modern cultivar	TaGW8-B1a
601	Taihemai3	Modern cultivar	TaGW8-B1a
602	Zhengke6	Modern cultivar	TaGW8-B1a
603	Jiamai6	Modern cultivar	TaGW8-B1b
604	Zhengmai518	Modern cultivar	TaGW8-B1b
605	Yanke316	Modern cultivar	TaGW8-B1b
606	Luomai2	Modern cultivar	TaGW8-B1b
607	Zhengnong5222	Modern cultivar	TaGW8-B1b
608	Liangmai958	Modern cultivar	TaGW8-B1b
609	Luomai32	Modern cultivar	TaGW8-B1b
610	Yanmai68	Modern cultivar	TaGW8-B1b
611	Mengnong1	Modern cultivar	TaGW8-B1b
